# Background pressure effects on MeV protons accelerated via relativistically intense laser-plasma interactions

**DOI:** 10.1038/s41598-020-75061-1

**Published:** 2020-10-26

**Authors:** Joseph Snyder, John Morrison, Scott Feister, Kyle Frische, Kevin George, Manh Le, Christopher Orban, Gregory Ngirmang, Enam Chowdhury, William Roquemore

**Affiliations:** 1 Department of Mathematical and Physical Sciences, Miami University, Hamilton, OH 45011 USA; 2 Innovative Scientific Solutions, Inc., Dayton, OH 45459 USA; 3 Department of Computer Science, California State University Channel Islands, Camarillo, CA 93012 USA; 4 Department of Physics, The Ohio State University, Columbus, OH 43210 USA; 5 Intense Energy Solutions, LLC, Plain City, OH 43064 USA; 6 Department of Material Science and Engineering, The Ohio State University, Columbus, OH 43210 USA; 7 Department of Electrical and Computer Engineering, The Ohio State University, Columbus, OH 43210 USA; 8 Air Force Research Laboratory, Dayton, OH 45433 USA

**Keywords:** Laser-produced plasmas, Plasma-based accelerators

## Abstract

We present how chamber background pressure affects energetic proton acceleration from an ultra-intense laser incident on a thin liquid target. A high-repetition-rate (100 Hz), 3.5 mJ laser with peak intensity of $8 \times 10^{18}\,\text {Wcm}^{-2}$ impinged on a 450 nm sheet of flowing liquid ethylene glycol. For these parameters, we experimentally demonstrate a threshold in laser-to-proton conversion efficiency at background pressures $< 8\,\text {Torr}$, wherein the overall energy in ions $>1\,\text {MeV}$ increases by an order of magnitude. Proton acceleration becomes increasingly efficient at lower background pressures and laser-to-proton conversion efficiency approaches a constant as the vacuum pressure decreases. We present two-dimensional particle-in-cell simulations and a charge neutralization model to support our experimental findings. Our experiment demonstrates that high vacuum is not required for energetic ion acceleration, which relaxes target debris requirements and facilitates applications of high-repetition rate laser-based proton accelerators.

## Introduction

Ultra-intense laser driven ions have potential applications in neutron production^1–3^, medical therapies and diagnostics^4,5^, and warm dense matter^6–8^, prompting numerous studies on the fundamental nature of ion acceleration with relativistic intensity lasers. Increasing the repetition rate of the source moves laser-driven ions closer to practical applications; however, there are still many challenges to overcome in terms of ion energy, flux, beam divergence, and development of a suitable high-repetition compatible target. Potential high-repetition rate targets for MeV ion acceleration include tape drive targets^9^, cryogenic targets^10^, and freestanding liquid crystal targets^11,12^. Flowing liquid targets are a promising prospect that scales well to multi-kHz operation^13^, although there are often issues with vapor pressure that lead to an increase in chamber pressure.

One of the predominant mechanisms currently studied for laser-driven ion acceleration is target normal sheath acceleration (TNSA)^14–16^. In TNSA, the laser deposits energy into hot electrons^17^ that subsequently generate quasi-static electric fields, ultimately driving ion acceleration^18–20^. There have been numerous experimental studies on the dependence of the ion energy spectra on laser parameters such as focal spot size, laser energy, and pulse width as well as target parameters, including material, thickness, and structured interfaces^21–29^. These efforts are often conservatively performed at very low pressures (e.g. $\sim 10^{-6}\,\text {Torr}$) in order to limit nonlinear effects on the laser pulse, reduce damage of optics, and facilitate high-voltage diagnostics. For cost effective application development, lowering the high vacuum requirement would be significant. Computational studies using particle-in-cell (PIC) simulations have further investigated TNSA across similar parameters mentioned above^30–32^. In these simulations, background gas is commonly neglected to simplify the simulation and minimize computational time. Thus, the effect of background gas on TNSA has been the subject of limited studies^33–37^.

We present an experimental study on the effect of background pressure on laser-driven ions in the TNSA regime. Above $\sim 8 \,\text {Torr}$ with our experimental conditions, the background gas degrades TNSA ion acceleration even in the case where the laser contrast is sufficient to not expand the target, which is known to limit TNSA sheath fields. We show proton acceleration becomes increasingly efficient at lower background pressures and laser-to-proton conversion efficiency approaches a constant as the vacuum pressure decreases. Below $\sim 1 \,\text {Torr}$, the conversion efficiency is within a few percent of that found at two orders of magnitude lower chamber pressure. A charge neutralization model and two-dimensional PIC simulations show good qualitative agreement with our experimental results. Our demonstration of multi-MeV ion acceleration using liquid targets and variable background pressures is, to our knowledge, the first thorough study of its kind. These results show MeV ion acceleration can readily be achieved with background pressures much higher than previously reported.

## Results

### Experimental results

**Figure 1:**
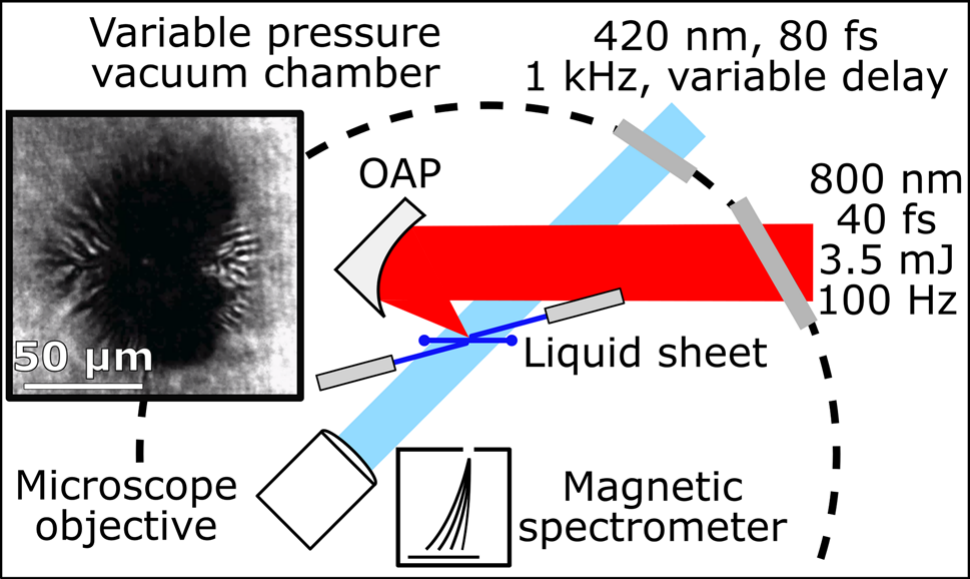
Experimental setup. After focusing, 3.5 mJ of laser energy is focused onto a 450 nm thick liquid target. Variable delay shadowgraphs are recorded using an 80 fs pulse timed to the high intensity pulse. The shadowgraphs reveal a 50–$60\,\upmu \text {m}$ radius ionization spot (inset).

**Figure 2:**
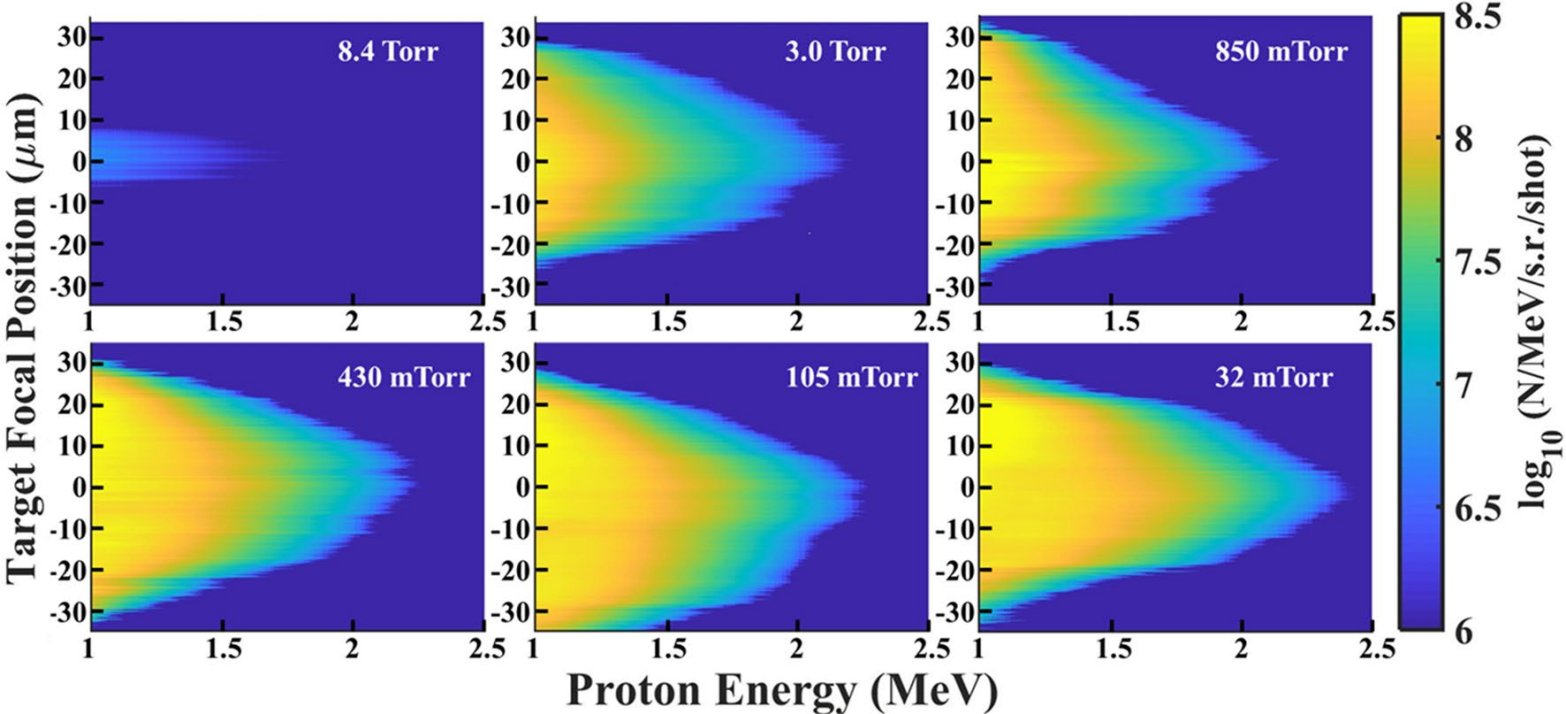
Proton energy spectra from a focal scan at different background pressures. The x-axis gives the proton energy in MeV, the y-axis shows the focal position of the target with 0 being set at the peak proton energy, and the color scale gives the proton number (N/MeV/s.r./shot) on a log scale.

Background chamber pressure was reduced from 13.7 Torr in discrete steps, and at each step, a series of shots while changing the position of the laser focus relative to the target were taken. The vacuum chamber setup is shown in Fig. 1. With 3.5 mJ on target, the peak intensity of the laser at best focus was $8 \times 10^{18}\, \text {Wcm}^{-2}$, well within the relativistic regime of laser-plasma interactions. Figure 2 shows the proton spectra results from performing the intensity scan at six different background pressures. Additional data was collected at 13.7 Torr chamber pressure, but there was no detectable $>1\, \text {MeV}$ ions on this scale. We noted no detectable change to the focal spot until the background pressure approached $\sim 80\,\text {Torr}$. The target position was moved along the laser propagation direction in $1.0\,\upmu \text {m}$ intervals such that 10 energy spectrum traces were recorded per target position with each trace integrated over 30 laser shots. The spectra were then normalized per shot. Scanning through focus allowed us to establish the peak focus and ensure appropriate prepulse conditions throughout all measurements. The $z=0$ position (best focus) was determined by selecting the trace with the highest proton cutoff energy as the zero point. Reduced laser energy and contrast improvements negated proton energy decrease at best focus caused by laser prepulse as seen previously in Morrison et al.^37^.

**Figure 3:**
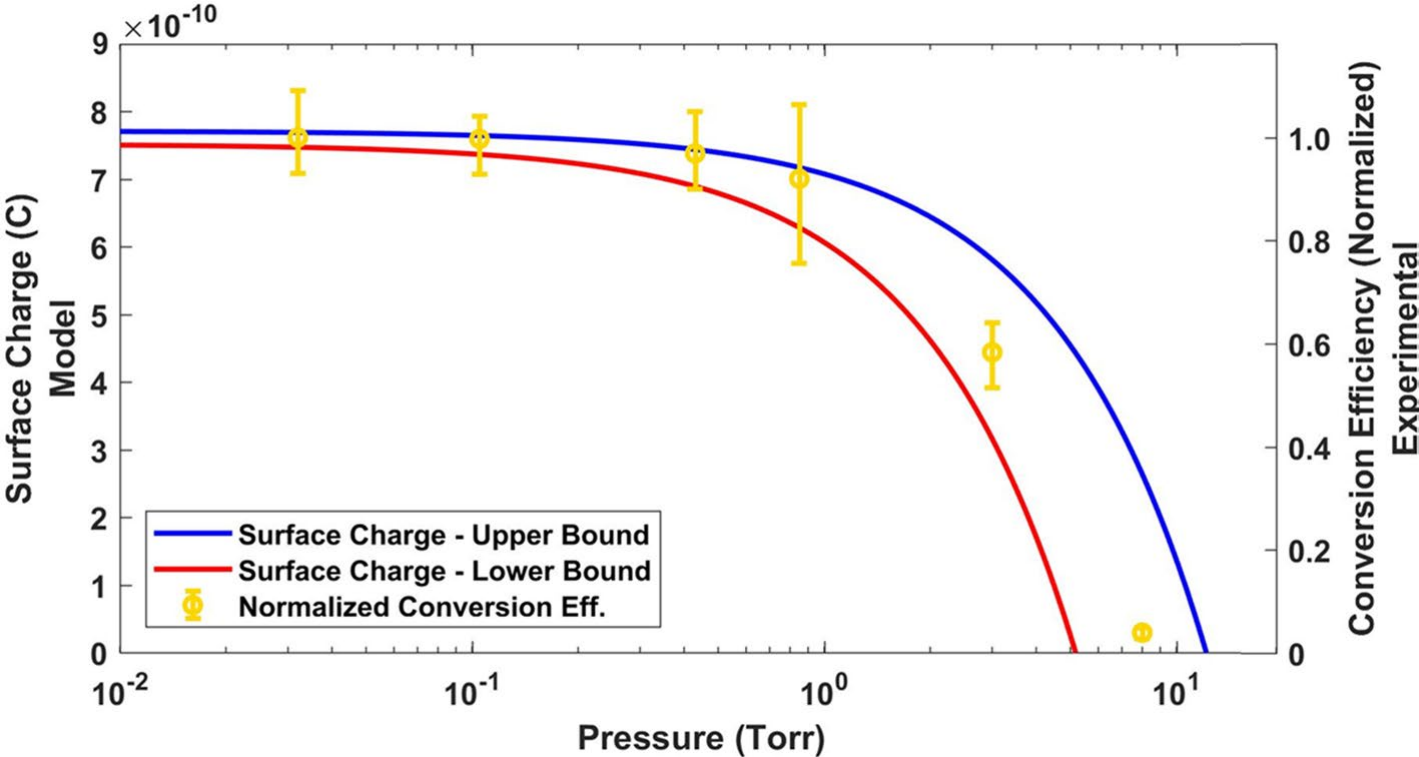
Normalized conversion efficiency and charge vs. pressure. The yellow data points show the amount of energy contained in energetic ($> 1\,\text{MeV}$) protons at best focus normalized to that found at 0.032 Torr background pressure. The predicted target charge from our model is shown in the solid lines where the upper/lower bound (blue/red) is the where the most/least background gas is needed to neutralize the target.

The spectra show a clear enhancement in conversion efficiency to high energy protons when the background pressure decreases (see also Fig. 3) with more than an order of magnitude increase in conversion efficiency as the pressure decreases from 8.4 to 3.0 Torr. Further, we note an asymptotic behavior of the conversion efficiency as the pressure decreases. When going from 0.430 to 0.032 Torr, more than an order of magnitude decrease in chamber pressure, the conversion efficiency is only increased by $\sim 3\%$. These results indicate that with our parameters, there is less than a $10\%$ difference in conversion efficiency below 0.850 Torr.

### Sheath field suppression model

To explain the improvement in conversion efficiency as the chamber pressure decreases below 8.4 Torr, we consider the effect of background gas on the accelerating sheath field. In the standard TNSA model, the laser accelerates electrons to MeV energies, which subsequently propagate and circulate through the target. The highest energy electrons move far away from the target region, leaving a positively charged region of the target which the remaining hot electrons turn back towards. These electrons reflux through the target and create a quasi-static electric field that accelerates the ions. The longitudinal spatial extent of this charge separation is estimated as the Debye length given by $\lambda _D = \left( \epsilon _0 k_B T_h/n_h e^2 \right) ^{1/2}$ where $k_B T_h\simeq 0.511 \left( \left( 1+\frac{I \lambda _0^2}{1.37 \times 10^{18} \text{W}\upmu \text{m}^2 \text{cm}^{-2}}\right) ^{1/2}-1 \right)$, is the hot electron temperature in MeV with *I* being the laser intensity in $\text {Wcm}^{-2}$, $\lambda _0$ the laser wavelength in μm, $\epsilon _0$ the permittivity of free space, $k_B$ the Boltzmann constant, $n_h$ the initial hot electron density, and *e* the fundamental charge unit^38^. The initial hot electron density $n_h$ is estimated by considering the number of hot electrons, $N_h$, that exist near the focal spot as $n_{h} = \frac{N_{h}}{\pi \left( \frac{s}{2} \right) ^2 \left( 2\lambda _D + d_t \right) }$^30^ where $N_h = \eta E_L/k_B T_h$, *s* is the FWHM intensity focal spot diameter of the laser, $d_t$ is the target thickness, $\eta = (1.2\times 10^{-15} (I\lambda _0^2)^{3/4})$ is the laser-to-hot-electron conversion efficiency for intensities between $10^{16}$ and $10^{19} \;\text{Wcm}^{-2}$^39,40^, and $E_L$ is the laser pulse energy. Using the laser parameters for our experiment, $I_{avg}=3.0\times 10^{18}$ $\text {Wcm}^{-2}$ ($I_{peak}=8.0\times 10^{18}\,\text {Wcm}^{-2}$), we find $k_B T_h \simeq 280\,\text {keV}$ (600 keV), $\eta \simeq 0.062$ (0.13), $\lambda _D \simeq 78 \,\text {nm}$ (120 nm), and $n_h \simeq 2.5\times 10^{21}\, \text {cm}^{-3}$ ($2.1\times 10^{21}\,\text {cm}^{-3}$).

The majority of hot electrons excited from the initially neutral target are trapped by the positive charge that maintains charge neutrality. These hot electrons recirculate through the target region because of the positive bulk charge, generating the necessary fields for ion acceleration. Without background gas, we estimate the total ion charge number by noting the charge neutrality condition $N_i = N_h$, where $N_i$ is the number of ions. When considering the vacuum chamber pressure, we note that with a liquid target, a background gas of evaporating liquid (in our case, ethylene glycol) fills a volume around the target. As the hot electrons confined within $\lambda _D$ from the target overtake the background gas, the resulting electric field will ionize the gas, providing cold electrons that act to cancel the positive charge of the target. Based on these assumptions, we estimate the total charge responsible for accelerating the ions when a background gas is present as $eN_{tot} = e(N_i - N_{e,bkg})$ where $N_{e,bkg}$ is the number of freed electrons in the background gas. Our model is qualitatively similar to that provided by Batani et al.^41^ to explain hot electron expansion velocity in a gas and Sokollik et al.^42^ to explain decrease charge from microsphere laser-target interactions, albeit the background pressure used in these works was far from the threshold demonstrated in this work.

The number of freed electrons from the background gas is found by assuming the volume in which background gas ionization occurs to be a cylinder with an effective radius, $r_{s}$, of the source size of background electrons and a height $2\lambda _D$, signifying the local extent of the electric field that would ionize the gas, where the factor of two recognizes the gas is present on both sides of the target. Using the ideal gas law, we estimate the number of molecules of gas within this effective volume $N_{gas}=\frac{2 P \pi r_{s}^2 \lambda _D}{k_B T_{bkg}}$ with $T_{bkg}$ being the ambient temperature of the gas ($\sim 300\,\text {K}$). Based on shadowgraphic images taken during the experiment (Fig. 1), we estimate $r_s \simeq 55 \pm 5\,\upmu \text{m}$, a reasonable assumption based on ion source size reported in literature^43,44^. We can estimate the peak strength of the electric sheath field ($E_s$) as $E_s \sim \frac{k_B T_h}{e \lambda _D}$^45^. Using this, $E_s \simeq 3.6{-}4.8\,\text {MV}\upmu \text {m}^{-1}$, which is similar to that found in simulations here and elsewhere^37^. With this field strength, it is reasonable to assume that over the ionization region, all components of the background gas surrounding the target will be singly ionized on average, suggesting a background electron number $N_{e,bkg} = Z N_{gas}$, where Z is the sum of all constituent atoms of the molecule. In our model, we assume the background gas nearest the target is ethylene glycol ($\text{C}_2 \text{H}_6 \text{O}_2$), therefore $Z=10$ (see “Methods” for additional considerations). Using the definitions above, we find the total charge of the target when considering background gas to be:

1 $$\begin{aligned} eN_{tot} = e\left( \frac{\eta _h E_L}{k_B T_h} - 2 \frac{Z P \pi r_{s}^2 \lambda _D}{k_B T_{bkg}}\right) \end{aligned}$$

The charge found in Eq. (1) is responsible for accelerating ions. As the background pressure increases, the overall target charge decreases, resulting in a reduced conversion efficiency to the laser accelerated ions. The total charge based on this model using our experimental parameters is shown in Fig. 3, where the upper bound assumes the lower bound on intensity ($I_{avg}=3.0\times 10^{18}\,\text{Wcm}^{-2}$) and the lower bound on electron source size ($r_s = 50\,\upmu \text{m}$) and the lower bound assumes the upper bound on intensity ($I_{peak}=8.0\times 10^{18}\,\text{Wcm}^{-2}$) and the upper bound on electron source size ($r_s = 60\,\upmu \text{m}$). The model results are co-plotted with the normalized conversion efficiency to target normal ions found experimentally at best focus. Based on the laser parameters used in the work, we expect the background gas to fully neutralize the accelerating fields when the pressure reaches between 5.2 and 12.2 Torr, in reasonable agreement with our experimental data. Our model predicts the total accelerating charge being 99% of its vacuum level at $\sim 50\,\text {mTorr}$, suggesting there should be little difference in conversion efficiency as the pressure decreases beyond this point.

### Simulation results

**Figure 4:**
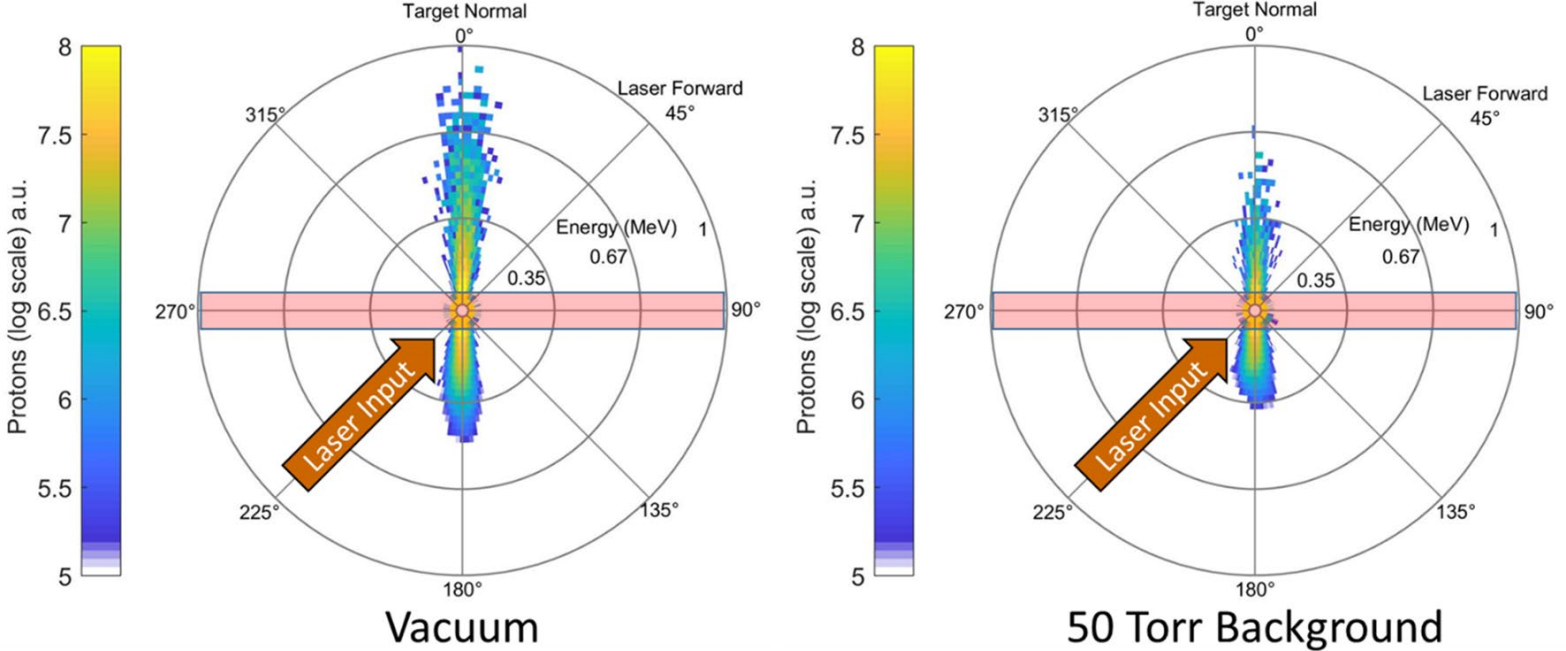
Simulation results comparing when the region surrounding the target is vacuum (left) and a 50 Torr background gas (right). The laser enters the simulation at a $45^\circ$ angle and interacts with a singly ionized ethylene glycol target that lies on the $90^\circ$–$270^\circ$ axis. The colorscale gives the number of protons in arbitrary units on a log scale. The position of a bin around the circle corresponds to the momentum angle of the proton 440 fs after the peak of the pulse reaches the target. The distance of the bin from the center of the circle gives the energy. In the vacuum case, the cutoff energy is $\sim 1 \,\text {MeV}$.

We have performed particle-in-cell (PIC) simulations to model the effects of background pressure on proton acceleration. The simulations were performed using VLPL^46^ (Virtual Laser Plasma Lab) in a two-dimensional configuration including a semi-classical ionization model^47–49^. Figure 4 shows the proton energy spectra 440 fs after the peak of the pulse reaches the front of the flat-top density of the target. In the vacuum case (left), the ion energy reaches 1.0 MeV, similar to that found experimentally. The difference in experimental cut-off energy and that found in the simulation is likely a result of incorrect pre-plasma conditions or abbreviated acceleration time in the simulation. In the high background pressure case (right), the maximum energy is reduced and the total conversion to protons $>0.5\,\text{MeV}$ ($50\%$ of the peak energy in vacuum) is only $\sim 10\%$ of that found in the vacuum simulation. Simulations also provide supporting evidence for sheath field suppression caused by the background gas. 40 fs after the peak of the pulse reaches the flat-top density region of the target, the peak average value of the x-component of the quasi-static electric sheath field on the rear of the target near the interaction region is approximately twice as high in the vacuum case when compared to the background pressure case (see Supplementary Information).

The sheath field suppression model presented above predicts a greater reduction in conversion efficiency to protons at 50 Torr than found in the simulation; however, we note that the model assumes a three-dimensional background region and an extended region of background gas to neutralize the target. In order to reduce computational costs, our simulations were run in two-dimensions with a smaller transverse extent than what is required by the model. These differences contribute to the discrepancy between the experimental and simulation results.

## Discussion

The sheath field suppression model is best used to approximate the pressures at which the background gas limits ion acceleration and does not capture the time dynamics of the accelerating field. Since the highest energy ions are also the earliest ions to gain their energy, these ions are likely least affected by the background gas neutralizing the accelerating field. This observation is consistent with our experimental data in Fig. 2 which shows a cutoff energy that only varies by $\sim 15\%$ across a three order of magnitude scan in pressure where ion acceleration was present. The ions accelerated at later times, and as such the lower energy ions, are more likely to be affected by the target neutralizing. This fact would present itself in the conversion efficiency, making the data presented in Fig. 3 a reasonable comparison.

We have experimentally demonstrated MeV level ion acceleration with a mJ class laser at 100 Hz using liquid targets at a background pressure much higher than commonly used. We present a simple charge neutralization model and PIC simulations to support our experimental findings. The data presented in Fig. 2 demonstrates the benefits and capabilities of high repetition rate experiments. Once designed and installed, the data collection took less than 6 h. Each of the subplots in Fig. 2 contains roughly 700 energy spectra traces, with each trace containing 30 laser shots, therefore the data presented in Fig. 2 represents nearly 130,000 relativistic laser-target interactions. Using our experimental parameters, ion acceleration is suppressed at high background pressures ($> 8 \,\text {Torr}$). As the background pressure decreases, there is a slight increase in ion cutoff energy and an asymptotic approach to moderate conversion efficiency. The pressure threshold for ion acceleration is likely system dependent. Scaling the model to higher laser intensities should lead to a stronger sheath field, a larger ionization spot, and a shorter Debye length. Our estimates suggest that at increased laser intensities, ion acceleration suppression occurs at pressures where optical aberrations would interfere with the interaction. This work informs the requirements to achieve proton acceleration which considerably relaxes the background pressures necessary, giving more freedom over experimental choice of target, repetition rate, diagnostics and has potential for cost effective applications.

## Methods

### Experiment

The experiment was conducted using the Red Dragon laser housed within the Extreme Light Laboratory at Wright Patterson Air Force Base in Dayton, OH. The experimental setup is shown in Fig. 1. The Ti:sapphire laser (800 nm) delivers 3.5 mJ on target with a 40 fs pulse duration into a focal spot size of $< 2.0\,\upmu \text{m}$ FWHM, reaching an average intensity of $3 \times 10^{18}\,\text{Wcm}^{-2}$ and a peak intensity of $8.0\times 10^{18}\, \text{Wcm}^{-2}$. The prepulse contrast of the laser was measured to be at the $10^{-6}$ level on the picosecond-timescale ($\pm 100\,\text {ps}$). On the nanosecond-timescale, the prepulse contrast is measured to be better than $10^{-10}$ (detection limited), apart from a pulse replica that exists at $-6.1\,\text {ns}$. The ns-pulse replica was below the intensity threshold that would limit ion acceleration at peak focus as was previously seen in Morrison et al.^37^ Although the target and laser are capable of operating at 1 kHz, the experiment was run at 100 Hz (250 Hz for 8.4 Torr data) for this study due to radiation exposure safety limits. The laser is focused by a F/1 protected gold coated off axis parabola onto a continuously flowing, 450 nm liquid sheet target^13^ at a $45^{\circ }$ angle of incidence. Target thickness was determined by the pressure and flow rate of the high pressure backing lines as well as the impact angle of the liquid microjets. The target has previously been characterized across the background pressures presented in this study using a Filmetrics device as described in Morrison et al. and George et al.^13,37^. A compact magnetic spectrometer which collected ion spectra was located along the rear target normal direction 130 mm from the interaction region. Particles entered into a 0.13 T magnetic field via a 1.0 mm entrance slit and were collected by a linear CCD covered with $508\,\upmu \text {m}$ of RP408 plastic scintillator bonded with index matching epoxy. To block scattered light, the CCD was covered with 60 nm of Al deposition. Although the magnetic spectrometer does not distinguish between ion species, calibration of the ion signal using CR-39 and mylar differential filtering suggests that in the energy range of interest ($>1\,\text{MeV}$), the measured signal is dominated by protons and heavier ions do not contribute significantly to the measurement^37^.

While operating the laser-target interaction, the lowest achievable background pressure with our setup was 32 mTorr. At pressures below 1 Torr, we varied the chamber pressure by adjusting the vacuum pump aperture. To achieve background pressures above 3 Torr, we backfilled the chamber with air. At low repetition rates, backfilling with air would alter the local background gas significantly, changing the field suppression model presented above. However, using diffusion estimates for the target liquid expanding to a gas then diffusing into air, we estimate that the majority of the local (within $\sim \upmu \text{m}$ of the target surface) background gas is ethylene glycol with our laser repetition rate even when backfilling with air. Even so, assuming a 50/50 mixture of ethylene glycol and air, our model (with $Z \simeq 6$) would predict a cutoff pressure for ion acceleration between $\sim 9$ and $\sim 20\,\text {Torr}$, which fits our lower boundary pressure (peak intensity) estimate well. Additionally, at the highest pressures we consider the effects of the protons traveling through a global background gas of air as they make their way to the detector. At the highest pressure presented in this manuscript, the background gas is responsible for down scattering a 1.0 MeV proton to $0.93 \pm 0.2\,\text {MeV}$ at the magnets and $\sim 0.87\,\text {MeV}$ at the detector. Higher proton energies are less affected by the background gas, and as such, we conclude that down scattering in the background gas does not significantly contribute to the substantial reduction in conversion efficiency seen at pressures above $\sim 1\,\text {Torr}$.

### Simulation

Simulations were performed using the particle-in-cell code VLPL. The simulation space was $65 \lambda _0 \times 70 \lambda _0$ with cell sizes $0.02 \lambda _0 \times 0.08 \lambda _0$ in the $x \times y$ dimensions, respectively. The target consisted of singly ionized hydrogen, carbon, and oxygen in the constituent ratios of ethylene glycol. The initial electron temperature was set to $\sim 2\,\text{eV}$ with matching velocity conditions for the ions, and there were initially 8 particle per cell of each species. The target region consisted of a $0.5\lambda _0$ flat-top density region beginning $15\lambda _0$ from the left of the simulation box with an initial electron density $n_e = 60n_c$, where $n_c =m_e\omega _0^2/4\pi e^2$ is the critical electron density with $m_e$ the electron mass and $\omega _0$ the laser angular frequency. Given the contrast of the laser system on the picosecond-timescale, we included a $2\lambda _0$ exponential profile with a $0.5\lambda _0$ scale length on the front of the target in the simulation to mimic preplasma conditions and improve laser-to-plasma coupling, while a linear $0.1\lambda _0$ rear of the target reduces target heating into the lower density region beyond the rear of the target. In the vacuum case, the remainder of the simulation space was empty. In the background plasma case, a neutral gas with atomic constituents of cold ethylene glycol filled the remainder of the simulation space with a particle number determined using the ideal gas law at room temperature to match a 50 Torr chamber environment. A laser with a profile $a = a_0 e^{-(r^2/\sigma _0^2)}\sin ^2[\pi t/(2\tau _0)]$ linearly polarized in the simulation plane enters the simulation space from the left boundary at a $45^\circ$ angle with respect to the target surface, with $a_0 = eE_0/m_e\omega _0c$, where $E_0$ is the amplitude of the laser. For our simulation, $a_0 = 2$ and $\sigma _0$ and $\tau _0$ are chosen such that the FWHM intensity spot size is $2.2 \,\upmu \text{m}$ and the FWHM intensity pulse duration is 42 fs.

## Supplementary information

Supplementary Information.

## References

[1] Disdier L, Garconnet J, Malka G, Miquel J (1999). Fast neutron emission from a high-energy ion beam produced by a high-intensity subpicosecond laser pulse. Phys. Rev. Lett..

[2] Roth M (2013). Bright laser-driven neutron source based on the relativistic transparency of solids. Phys. Rev. Lett..

[3] Storm M (2013). Fast neutron production from lithium converters and laser driven protons. Phys. Plasmas.

[4] Borghesi, M. & Macchi, A. Laser-driven ion accelerators: State of the art and applications. In *Laser-Driven Particle Acceleration Towards Radiobiology and Medicine*, 221–247 (Springer, Berlin, 2016).

[5] Fritzler S (2003). Proton beams generated with high-intensity lasers: Applications to medical isotope production. Appl. Phys. Lett..

[6] Patel P (2003). Isochoric heating of solid-density matter with an ultrafast proton beam. Phys. Rev. Lett..

[7] Roth M (2009). Proton acceleration experiments and warm dense matter research using high power lasers. Plasma Phys. Control. Fusion.

[8] Bang W (2015). Uniform heating of materials into the warm dense matter regime with laser-driven quasimonoenergetic ion beams. Phys. Rev. E.

[9] Noaman-ul Haq M (2017). Statistical analysis of laser driven protons using a high-repetition-rate tape drive target system. Phys. Rev. Accel. Beams.

[10] Gauthier M (2016). High-intensity laser-accelerated ion beam produced from cryogenic micro-jet target. Rev. Sci. Instrum..

[11] Poole P (2016). Moderate repetition rate ultra-intense laser targets and optics using variable thickness liquid crystal films. Appl. Phys. Lett..

[12] Poole P (2014). Liquid crystal films as on-demand, variable thickness (50–5000 nm) targets for intense lasers. Phys. Plasmas.

[13] George, K. *et al.* High repetition rate ($$>=$$ khz) targets and optics from liquid microjets for the study and application of high intensity laser-plasma interactions. arXiv preprint arXiv:1902.04656 (2019).

[14] Snavely R (2000). Intense high-energy proton beams from petawatt-laser irradiation of solids. Phys. Rev. Lett..

[15] Clark E (2000). Measurements of energetic proton transport through magnetized plasma from intense laser interactions with solids. Phys. Rev. Lett..

[16] Wilks S (2001). Energetic proton generation in ultra-intense laser-solid interactions. Phys. Plasmas.

[17] Link A, Freeman RR, Schumacher D, Van Woerkom L (2011). Effects of target charging and ion emission on the energy spectrum of emitted electrons. Phys. Plasmas.

[18] Crow J, Auer P, Allen J (1975). The expansion of a plasma into a vacuum. J. Plasma Phys..

[19] Mora P (2003). Plasma expansion into a vacuum. Phys. Rev. Lett..

[20] Roth M, Schollmeier M (2016). Ion acceleration–target normal sheath acceleration. CERN Yellow Rep..

[21] Roth M (2002). Energetic ions generated by laser pulses: A detailed study on target properties. Phys. Rev. Special Top. Accel. Beams.

[22] Schwoerer H (2006). Laser-plasma acceleration of quasi-monoenergetic protons from microstructured targets. Nature.

[23] Lindau F (2005). Laser-accelerated protons with energy-dependent beam direction. Phys. Rev. Lett..

[24] Zani A, Sgattoni A, Passoni M (2011). Parametric investigations of target normal sheath acceleration experiments. Nucl. Instrum. Methods Phys. Res. Sect. A Accel. Spectrom. Detect. Assoc. Equip..

[25] Flippo K (2008). Increased efficiency of short-pulse laser-generated proton beams from novel flat-top cone targets. Phys. Plasmas.

[26] Schollmeier M (2008). Laser beam-profile impression and target thickness impact on laser-accelerated protons. Phys. Plasmas.

[27] Brenner C (2011). Dependence of laser accelerated protons on laser energy following the interaction of defocused, intense laser pulses with ultra-thin targets. Laser Particle Beams.

[28] Poole P (2018). Laser-driven ion acceleration via target normal sheath acceleration in the relativistic transparency regime. New J. Phys..

[29] Fuchs J (2006). Laser-driven proton scaling laws and new paths towards energy increase. Nat. Phys..

[30] Brenner C, McKenna P, Neely D (2014). Modelling the effect of laser focal spot size on sheath-accelerated protons in intense laser-foil interactions. Plasma Phys. Control. Fusion.

[31] Snyder J, Ji L, Akli K (2016). Enhancement of laser intensity and proton acceleration using micro-tube plasma lens targets. Phys. Plasmas.

[32] Esirkepov T, Yamagiwa M, Tajima T (2006). Laser ion-acceleration scaling laws seen in multiparametric particle-in-cell simulations. Phys. Rev. Lett..

[33] Perego M, Howell P, Gunzburger M, Ockendon J, Allen J (2013). The expansion of a collisionless plasma into a plasma of lower density. Phys. Plasmas.

[34] Baton S (2005). Recent experiments on electron transport in high-intensity laser matter interaction. Plasma Phys. Control. Fusion.

[35] Batani D (2009). Laser-driven fast electron dynamics in gaseous media under the influence of large electric fields. Phys. Plasmas.

[36] Tatarakis M (2003). Propagation instabilities of high-intensity laser-produced electron beams. Phys. Rev. Lett..

[37] Morrison JT (2018). Mev proton acceleration at khz repetition rate from ultra-intense laser liquid interaction. New J. Phys..

[38] Wilks S, Kruer W, Tabak M, Langdon A (1992). Absorption of ultra-intense laser pulses. Phys. Rev. Lett..

[39] Yu J, Jiang Z, Kieffer J, Krol A (1999). Hard x-ray emission in high intensity femtosecond laser-target interaction. Phys. Plasmas.

[40] Schreiber J (2006). Analytical model for ion acceleration by high-intensity laser pulses. Phys. Rev. Lett..

[41] Batani D (2005). Ultraintense laser-produced fast-electron propagation in gas jets. Phys. Rev. Lett..

[42] Sokollik T (2009). Directional laser-driven ion acceleration from microspheres. Phys. Rev. Lett..

[43] Borghesi M (2004). Multi-mev proton source investigations in ultraintense laser-foil interactions. Phys. Rev. Lett..

[44] Nürnberg F (2009). Radiochromic film imaging spectroscopy of laser-accelerated proton beams. Rev. Sci. Instrum..

[45] Macchi A, Borghesi M, Passoni M (2013). Ion acceleration by superintense laser-plasma interaction. Rev. Mod. Phys..

[46] Pukhov A (1999). Three-dimensional electromagnetic relativistic particle-in-cell code vlpl (virtual laser plasma lab). J. Plasma Phys..

[47] Ammosov M (1986). Tunnel ionization of complex atoms and of atomic ions in an alternating electric field. Sov. Phys. JETP.

[48] Karmakar A, Pukhov A (2007). Collimated attosecond gev electron bunches from ionization of high-z material by radially polarized ultra-relativistic laser pulses. Laser Particle Beams.

[49] Perelomov A, Popov V, Terent’ev M (1966). Ionization of atoms in an alternating electric field. Sov. Phys. JETP.

