# Supplementary material for: Background pressure effects on MeV protons accelerated via relativistically intense laser-plasma interactions
Source: Sci Rep. 2020 Oct 26;10:18245. doi: 10.1038/s41598-020-75061-1 (PMC7588495; doi:10.1038/s41598-020-75061-1)
Supplement: Supplementary file 1 — Supplementary Information. [file 41598_2020_75061_MOESM1_ESM.pdf]

Supplementary information

Background pressure effects on MeV protons accelerated via relativistically intense laser-plasma interactions

Joseph Snyder[1,*], John Morrison[2], Scott Feister[3], Kyle Frische[2], Kevin George[2], ManhLe[4], Christopher Orban[4], Gregory Ngirmang[4], Enam Chowdhury[5,6,7,4], and William Roquemore[8]

[1]Miami University, Department of Mathematical and Physical Sciences, Hamilton OH, 45011 USA
[2]Innovative Scientific Solutions, Inc., Dayton, OH 45459, USA
[3]California State University Channel Islands, Department of Computer Science, Camarillo, CA 93012, USA
[4]The Ohio State University, Department of Physics, Columbus, OH 43210, USA
[5]Intense Energy Solutions, LLC Plain City, OH, 43064 USA
[6]The Ohio State University, Department of Material Science and Engineering, Columbus, OH 43210, USA
[7]The Ohio State University, Department of Electrical and Computer Engineering, Columbus, OH 43210, USA
[8]Air Force Research Laboratory, Dayton, OH 45433, USA
*snyderjc@miamioh.edu

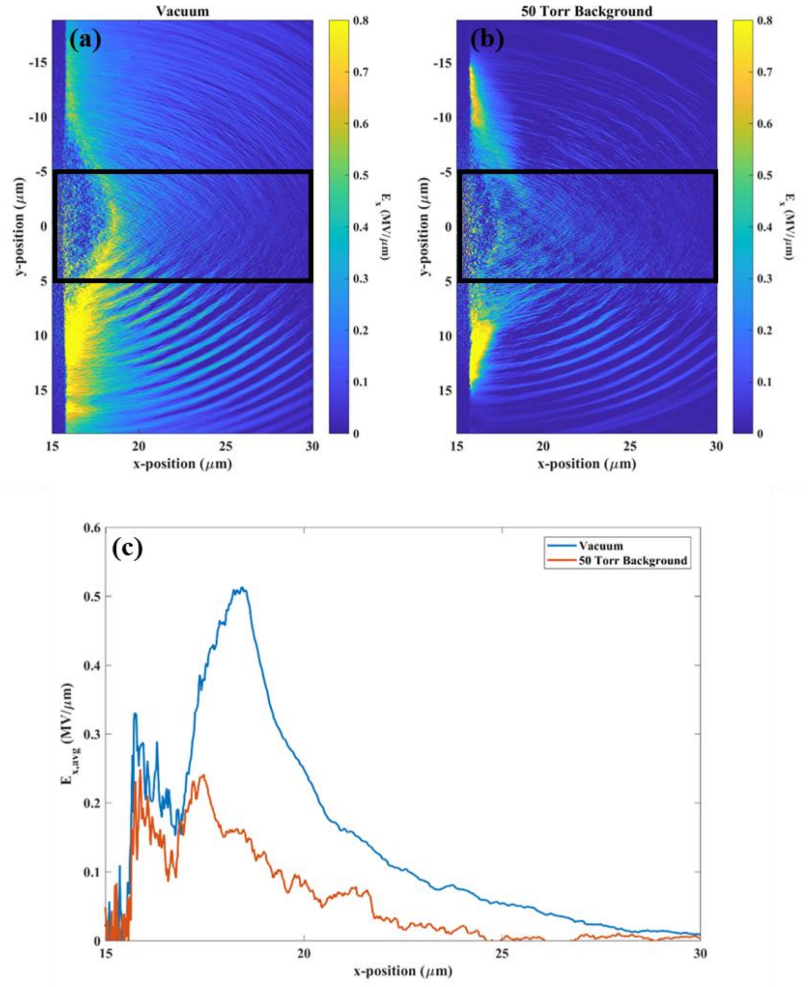

Supplementary Figure S1. E_x at the rear of the target extracted from the simulations 40 fs after the peak of the pulse reaches the flat top density region of the target in the case of (a) vacuum and (b) 50 Torr background. The laser interacts with the target at y = 0. In the background case, one can see the sheath field near the interaction region has deteriorated while the sheath field remains significantly stronger in the vacuum case. E_x value vs. x-position averaged from y = -5 to y = +5 (within the black boxes in (a) and (b)) is given in (c), providing evidence that the presence of the background gas significantly suppresses the sheath field that is responsible for accelerating ions.
